# Editorial: Increasing crop yield: the interaction of crop plant roots with their environment

**DOI:** 10.3389/fpls.2023.1249270

**Published:** 2023-08-28

**Authors:** Saoirse Tracy, Hannah Schneider, Josefine Kant

**Affiliations:** ^1^ School of Agriculture and Food Science, University College Dublin, Dublin, Ireland; ^2^ Centre for Crop Systems Analysis, Wageningen University & Research, Wageningen, Netherlands; ^3^ Institute for Bio- & Geosciences 2, Forschungszentrum Jülich GmbH, Jülich, Germany

**Keywords:** roots, crops, yield, abiotic stress, GxE

The quest for global food security has long been a pressing challenge, as the world’s population continues to grow. One crucial aspect of addressing this challenge lies in maximizing crop yields through a deeper understanding of the intricate relationship between crop plant roots and their environment. Root systems play a fundamental role in nutrient uptake, water absorption, and anchoring plants into the soil. Recent advancements in agricultural research have shed light on the dynamic interactions occurring beneath the surface, uncovering the potential to enhance crop productivity and sustainability. In this Research Topic, we delve into the fascinating world of root-environment interactions and explore the latest breakthroughs and innovative approaches that hold promise for revolutionizing agricultural practices and meeting the demands of an ever-expanding population. Crop roots support establishment, development, and growth of plants that are the basis of human nutrition. To value the importance of crop root research, this Research Topic invited manuscripts of crop plants such as maize, rice, wheat, potato, soybean, and others summing up to four original research articles, two systematic review articles and one review article.

The papers represent an eclectic mix that demonstrates the range of research on interactions with crop root systems and their dynamic environment. The highlights include Mazarei et al.’s identification of two candidate genes in soybean through transcription profiling, *GmNAC19* and *GmGRAB1.* When overexpressed, *GmNAC19* and *GmGRAB1* resulted in greater root length and root biomass and seed yield doubled. In addition, overexpression of *GmNAC19* resulted in greater drought tolerance. Similarly, a systematic review by Lopez et al. focused on how nutrient deficiencies, including nitrogen, phosphorus, and potassium, in arable crops affected root development. In a meta-analysis of fifty field experiments, root length and root biomass were reduced while root length per shoot biomass increased in nitrogen and phosphorus deficiency. The root to shoot ratio was increased in nitrogen deficiency. As a highlight of how roots play an important role in aboveground plant growth and performance, Zheng et al. performed a systematic review on drought stress through a bibliometric analysis from the last century. Historically, research on aboveground organs has dominated the literature. However, recently root research is getting more attention with the specific topics of root plasticity in drought and drought recovery emerging as large new areas of interest. The authors also highlight and summarize root responses to different phases of drought and stress recovery.

Root responses to soil compaction are also emerging as an important Research Topic. Nawaz et al. describe root responses to soil compaction by field traffic on a maize field with sandy loam soil. Soil compaction caused by field traffic resulted in a shallow and denser hard pan in the top soil and subsequently greater root growth in the top 10 cm of soil and less growth between 10 to 30 cm soil depth. Compared to the non-trafficked control, genotypic differences were observed in root growth and yield reduction. Similarly, Skilleter et al. described responses of two contrasting potato cultivars to soil compaction. One genotype was relatively responsive to compaction stress and had reduced leaf area, root density, and yield, while the other genotype had less pronounced responses to compaction. The compacted soils generally contained more water and had a lower soil resistance.

In addition to compaction stress, plants also experience a wide range of other abiotic stresses. Sheldon and Munns reviewed root plasticity responses to salinity stress in staple cereal crops including wheat, maize, rice and barley. Cereal crops are usually classified as glycophytes, meaning salt-sensitive. However, variation in salt-tolerance within and between species is observed. They proposed a new ideotype for salt tolerance to reduce sodium uptake and minimize transport including a ligninfied layer in the root tissue, more lateral roots, deeper rooting, and larger cortical cells. Ning et al. also examined both root anatomical and architectural traits and described how IAA treatment enhances and accelerates root cortical aerenchyma formation. They used root rotation as a gravitropic stimulus to alter lateral root formation on the convex root side. This is a result of the accumulation of auxin in the pericycle of the convex root side resulting in the formation of more lateral roots but less and slower aerenchyma formation in rotated crown roots. Laser microdissection of cortical tissue from both concave and convex sides showed auxin-responsive gene expression decreased on the convex side of the root.

With the seven papers in this Research Topic on *Increasing Crop Yield: The Interaction of Crop Plant Roots with Their Environment*, we hope the readers can gain a better appreciation of the intricacies of root interactions to the dynamic soil environment and how these responses influence plant growth and yield. The exploration of crop plant root-environment interactions has illuminated the path towards sustainable agriculture and global food security. The papers presented in this Research Topic collectively emphasize the critical role of root systems in supporting crop growth and call for continued research and innovation to grow our understanding of the function and genetic control of roots. While understanding and optimizing root systems hold great promise for the development of more productive crops that require less inputs, including fertilizer and irrigation, and that are more resilient to stress, the measurement and manipulation of root traits is challenging. The role of roots in plant function, growth, and ultimately yield performance depends on a number of factors including the choice of crop, soil properties, climatic conditions, management practices, and interactions among and between root and shoot traits. The investigations of root responses to environmental factors sheds light on the need for tailored management practices and the importance of root traits as tools to mitigate yield losses. Yield stability or increase in yield potential will be aided by engaging cutting-edge technologies like precision agriculture, advanced breeding techniques, and genomics to optimize root systems to local environments. By unraveling the secrets of root-environment interactions, we can pave the way for a sustainable and food-secure future.

## Author contributions

JK, HS, and ST jointly wrote the manuscript, revised the text and approve of its publication.

